# Clinical Applications of Fractional Flow Reserve Derived from Computed Tomography in Coronary Artery Disease

**DOI:** 10.1016/j.mcpdig.2024.100187

**Published:** 2024-12-14

**Authors:** Cappi Chan, Min Wang, Luoyi Kong, Leanne Li, Lawrence Wing Chi Chan

**Affiliations:** a Department of Health Technology and Informatics, Hong Kong Polytechnic University, Hong Kong, China; b Key Laboratory for Biomedical Engineering of Ministry of Education, College of Biomedical Engineering and Instrument Science, Zhejiang University, Hangzhou, China; c School of Medicine, Sir Run Run Shaw Hospital, Department of Endocrinology, Zhejiang University, Hangzhou, Zhejiang, China; d Medical Systems Division, FUJIFILM Hong Kong Limited, Tseun Wan, Hong Kong

## Abstract

Computer tomography–derived fractional flow reserve (CT-FFR) represents a significant advancement in noninvasive cardiac functional assessment. This technology uses computer simulation and anatomical information from computer tomography of coronary angiogram to calculate the CT-FFR value at each point within the coronary vasculature. These values serve as a critical reference for cardiologists in making informed treatment decisions and planning. Emerging evidence suggests that CT-FFR has the potential to enhance the specificity of computer tomography of coronary angiogram, thereby reducing the need for additional diagnostic examinations such as invasive coronary angiography and cardiac magnetic resonance imaging. This could result in savings in financial cost, time, and resources for both patients and health care providers. However, it is important to note that although CT-FFR holds great promise, there are limitations to this technology. Users should be cautious of common pitfalls associated with its use. A comprehensive understanding of these limitations is essential for effectively applying CT-FFR in clinical practice.

## Article Highlights

• Computer tomography–derived fractional flow reserve is an algorithm that processes computer tomography of coronary angiogram (CTCA) images to model the fluid dynamics of coronary arteries. It provides functional information to assess the hemodynamic impact caused by coronary artery stenosis.
• Computer tomography–derived fractional flow reserve improves the specificity of CTCA in assessing lesion-specific ischemia. It acts as a gatekeeper to exclude nonobstructive coronary artery disease in invasive coronary angiogram.
• Techniques such as artificial intelligence and reduced-order computation could accelerate computation. Algorithms with enhanced speed, accuracy, and lower cost are being developed.

Computed tomography (CT) has been widely used to assess coronary artery disease (CAD) in patients with chest pain. It is a noninvasive, readily available, and cost-effective imaging modality in ruling out CAD. Before CT, an invasive coronary angiogram (ICA) was used to assess CAD and has been the gold standard in this field. Technological advancement in CT scanners makes cardiac imaging possible and more accurate, matching the accuracy of ICA. High negative predictive value has been reported by research, making it an effective tool in screening CAD.

Conventionally, when severe obstruction of the coronary artery is detected in computed tomography of coronary angiogram (CTCA), referral to a cardiac catheterization laboratory for revascularization treatment would be considered. If lesion-specific ischemia is uncertain, fractional flow reserve (FFR) could be measured before stenting the lesion. It was suggested that FFR of less than or equal to 0.8 indicates stenosis with hemodynamic impact, which favors the decision of revascularization. Percutaneous coronary intervention (PCI) with a drug-eluting stent would often be performed. When FFR is more than 0.8, the stenosis shows an insignificant effect on its hemodynamics. Optimal medical therapy (OMT) is recommended to control the progression of CAD.

During the interventional procedure, FFR could be selectively measured by using guidewire sensor technology. By placing the wire with a sensor across the plaque, coronary blood pressure, flow velocity, and resistance could be measured proximal and distal to the lesion. Fractional flow reserve is the ratio of maximum blood flow distal to the lesion to that of proximal to the lesion. Hyperemia is induced by injecting adenosine intravenously or intracoronary via the catheter. The pressure gradient is then measured to calculate the ratio.

It is important to decide which stenosis or patient requires revascularization. Despite luminal stenosis observed in CTCA, some do not necessarily cause hemodynamic impact (ie, ischemia of the supplying myocardium). There would occasionally be discordant results between anatomical stenosis and functional impact. Multiple factors could affect the hemodynamics and risk of ischemia, including minimal luminal diameter, characteristics of the lesion, the myocardial mass of the patient, reference vessel size of proximal and distal segments, and whether the lesion is diffuse or focal. The severity of stenosis may not be the sole determinant in patient management. For instance, mild but diffuse stenosis and lesions in the proximal segment could contribute to more ischemia. One study investigated the diagnostic accuracy of 64-slice CT using FFR as standard. It was found that only 49% of the detected stenosis caused FFR of less than or equal to 0.75.^1^ Owing to the high false-positive rate of CTCA, further assessment of the patient with positive findings is recommended to guide revascularization. Revascularizing the artery that has FFR of more than 0.8 does not yield a better prognosis and may be deemed inappropriate.

**Figure 1:**
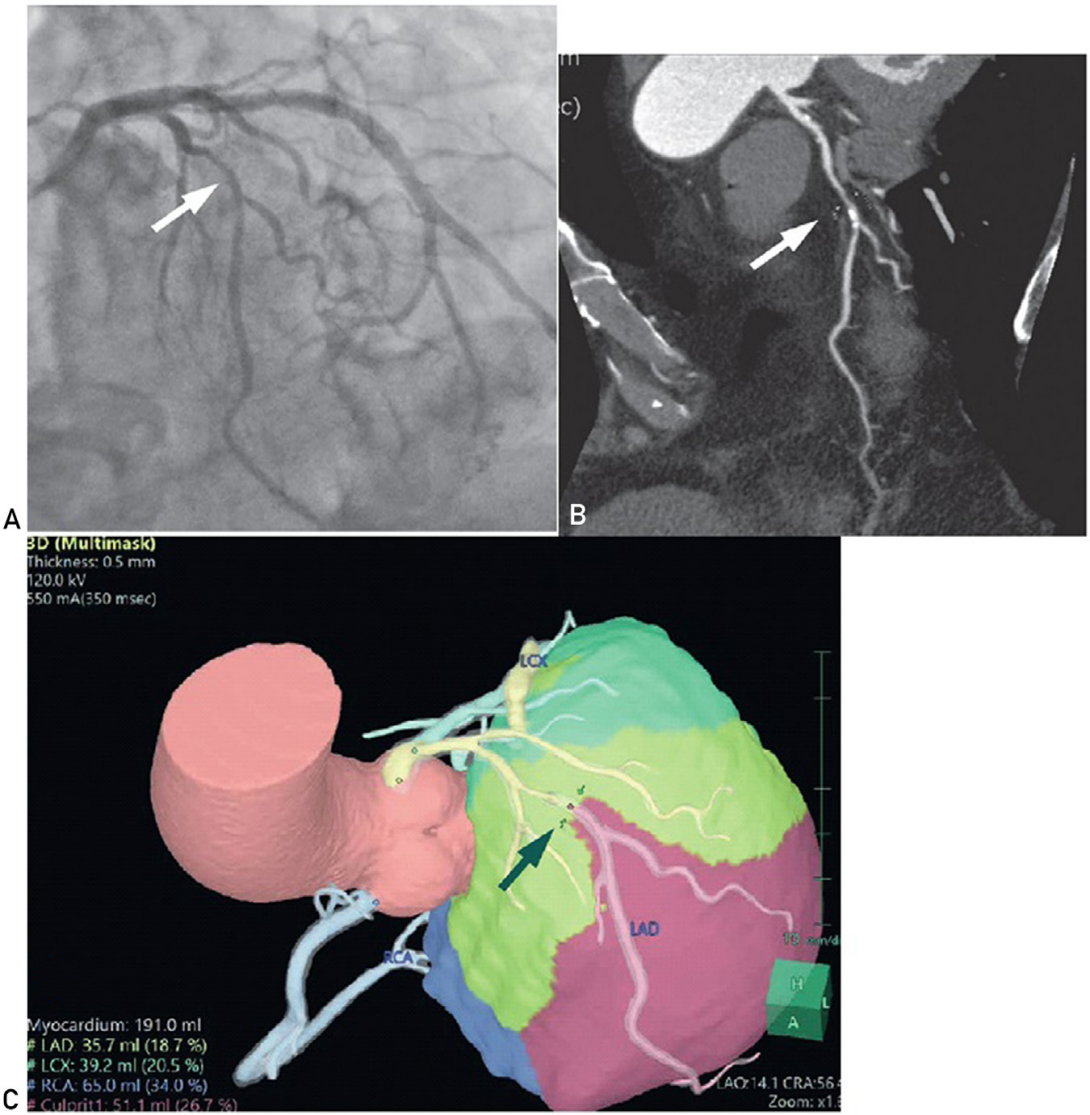
Invasive coronary angiogram (A) and computer tomography of coronary angiogram (B) found a calcified lesion (arrows) at the midsegment of the left anterior descending artery (LAD); the minimal lumen diameter was 1.4 mm. C, After setting a specific point at the culprit lesion (arrow), the software program (Synapse Vincent; Fujifilm) found that the myocardial mass at risk (MMAR) was 51.1 mL and that the %MMAR was 26.7%. LCx, left circumflex artery; RCA, right carotid artery.

Although measurement of FFR is necessary to prevent unnecessary treatment, the associated cost and risk should be considered. Alternatives have been proposed to circumvent the dependence on invasive FFR. These include but are not limited to computer tomography–derived fractional flow reserve (CT-FFR) and myocardial mass at risk (MMAR). Both methods improve diagnostic accuracy by analyzing CTCA images without further examination or imaging. They have been reported to yield better accuracy in detecting lesions with hemodynamic significance.^1^^,^^2^ Take MMAR as an example, standard Digital Imaging and Communications in Medicine data were transferred to a 3-dimensional (3D) imaging workstation incorporating the novel software program (Synapse Vincent; Fujifilm). A 3D reconstruction of the coronary arteries and left ventricular myocardium was made after automatic and additional manual tracing of all visible coronary branches. Myocardial territory was calculated using an algorithm on the basis of Voronoi tessellation. After setting a specific point at the culprit lesion in the coronary artery tree, the corresponding myocardial volume, that is, MMAR, was calculated by including all voxels distal to the point and closer to the compromised coronary artery than any others. The %MMAR was then calculated automatically as the percentage of the corresponding myocardium regarding the total left ventricular myocardial mass. Figure 1 shows a representative case.

Conventionally, patients with moderate stenosis (>50%) would be referred to further diagnostics and determine the need for revascularization. Ongoing research aims to further enhance the diagnostic capabilities of CTCA. Computer-assisted analysis of CTCA potentially leads to a novel approach to functional assessment. CT-FFR is among the various technologies focused on automated functional analysis. In this article, the basic principle behind CT-FFR and its clinical application is discussed. This review also focuses on how CT-FFR could revolutionize contemporary diagnostic pathways and be beneficial to patients.

## Deriving the CT-FFR

The foundation of computational simulation for coronary blood flow was developed in 2010 and termed computational fluid dynamics (CFD). The key idea is to simulate the blood flow using incompressible Navier-Stokes equations. The key elements of the model also consider the interaction between the myocardium and the vessels, the traction force exerted by the arterial wall, and the hemodynamic impact of the stenosis. This method requires the computation of many partial differential equations for a finite time interval in 1 cardiac cycle. To obtain patient-specific data for simulation, CTCA is performed, and an anatomical model of the coronary tree is extracted. The finite-element mesh is applied to the segmented anatomical model. Once the vessel is identified, millions of elements in the geometric model are solved for velocity and pressure. The complex simulation requires a supercomputer to process. HeartFlow, the CFD model for CT-FFR measurement, has obtained FDA approval in 2022. The first step of the diagnosis pathway is data acquisition of CTCA. Data are then uploaded to the HeartFlow cloud server and processed by artificial intelligence and technicians before simulating fluid dynamics. Further, CT-FFR values would be calculated at each point of the coronary tree. The value at 2 cm distal to the target lesion was reported to yield better accuracy than the lowest value of the vessel.^3^ The physiologic impact of the stenosis can then be studied by the physician. It is claimed that most of the analysis can be completed within 5 hours.

Another approach to the problem is to use machine learning (ML) software. The basic principle of this approach is to train a deep learning model using synthetically generated coronary anatomies. The model is trained against CFD and learns to integrate the relationship between various features extracted from the synthetic anatomies. The trained model could identify vessel radius, length, and degree of tapering. The CT-FFR can therefore be derived from the model built from the database. This approach simplifies the computation process and can be completed on the onsite workstation. The correlation between CT-FFR_ML_ and CT-FFR_CFD_ was studied by Coenen et al.^4^ The results generated by these 2 approaches were found to have high reproducibility (Pearson *R*=0.997). A notable example is DEEPVESSEL FFR made by Keya Medical. It is an FDA-cleared product in 2022 that uses ML and imaging processing in cloud servers.

## The Evidence Supporting CT-FFR in Diagnosis of CAD and ITS Application

First, the addition of CT-FFR to CTCA could further improve its diagnostic accuracy, especially the specificity. One study retrospectively investigated the accuracy of CT-FFR_ML_ against ICA with FFR measurement between 2008 and 2017. This research investigated the accuracy of CT-FFR and various plaque characteristics in detecting ischemic stenosis. The plaque characteristics such as lesion length, plaque burden, remodeling index, presence of Napkin-ring sign, and degree of stenosis were studied. Of all these parameters, CT-FFR was shown to yield a better area under the curve (AUC) in the receiver operating characteristic curve shown in Supplemental Figure 1 (available online at https://www.mcpdigitalhealth.org/). Supplemental Table 1 (available online at https://www.mcpdigitalhealth.org/), shows that plaque characteristics generally lacked specificity and positive predictive value, indicating the possibility of overestimating the severity of CAD. It was established that CT-FFR of less than or equal to 0.80 was a reliable predictor of lesion-specific ischemia with improvement in specificity and positive predictive value (sensitivity, 82%; specificity, 94%; positive predictive value, 88%; negative predictive value, 92%).^5^ Thus, its better discriminatory power could improve CTCA as a gatekeeper to ICA by reducing the number of false-positive cases. This would minimize the chance of overestimating CAD, thus saving resources on follow-up examinations.

The diagnostic accuracy of CT-FFR on lesion-specific ischemia has been extensively studied. Coenen et al^4^ investigated the correlation between CT-FFR and invasive FFR. The study also supported the claim that CT-FFR could differentiate functionally significant stenosis. In this example, lesions with more than 50% stenosis in 382 vessels were selected and included. Two groups of 245 and 249 vessels were classified as hemodynamically significant obstructions by the ML model and CFD model of Siemens cFFR, respectively. The AUC of receiver operating characteristic curve was 0.84 for both approaches in detecting lesions with FFR of less than or equal to 0.8. Assessing the vessel, both CT-FFR approaches significantly improved specificity compared with CTCA with stenosis of more than 50% as criteria. Nevertheless, this study elucidated significant findings about the misuse of CT-FFR. First, CT-FFR and invasive FFR have a moderate correlation (Pearson *R*=0.62). Direct comparison between CT-FFR and FFR values would be deemed inappropriate. Second, the accuracy of CT-FFR was shown to fall markedly to 60% when the invasive FFR of the lesion was between 0.76 and 0.80, representing the borderline cases of lesion-specific ischemia. Another systemic review citing studies from 2011 to 2016 found that the accuracy of CT-FFR in the range of 0.70-0.80 was 46.1%, in concordance with invasive FFR.^6^ Although CT-FFR reported superior discriminatory power, improvement in accuracy is needed to further narrow down this gray zone. One example is xFFR designed by General Electric. A study published in 2023 claimed that it could achieve an AUC of 0.975, sensitivity of 92.9%, and specificity of 100% regarding invasive FFR.^7^ This shows a remarkable improvement in diagnostic performance. However, the performance in reality has yet to be seen because it is not commercially available. Supplemental Table 2 (available online at https://www.mcpdigitalhealth.org/) summarizes the diagnostic performance of common CT-FFR algorithms.^4^^,^^7^, ^8^, ^9^, ^10^ When the result falls into the borderline range and is inconclusive, individualized plans should be made regarding clinical background and anatomy.

**Figure 2:**
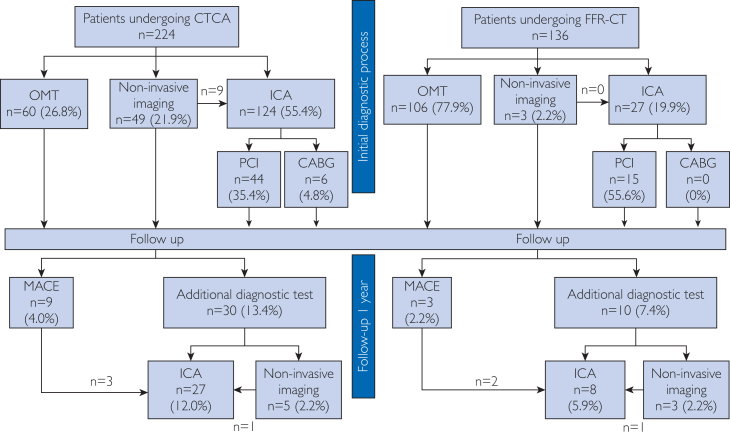
The clinical management and outcome after 1-year follow up in patients who underwent CTCA or CT-FFR analysis.^11^ The diagram is adapted from Becker et al.^11^ CABG, coronary artery bypass graft; CTCA, computer tomography of coronary angiogram; CT-FFR, computer tomography–derived fractional flow reserve; ICA, invasive coronary angiogram; MACE, major adverse cardiovascular event; OMT, optimal medical therapy; PCI, percutaneous coronary intervention.

Second, the implementation of CT-FFR in diagnostic procedures and subsequent patient management strategies does not compromise short-term patient survival outcomes or exacerbate complication rates. Compared with CT-FFR, conventional diagnostic strategies are more conservative and favor revascularization over OMT. In addition, CT-FFR considerably influences subsequent treatment strategies, potentially impacting clinical outcomes. A concern regarding the safety of CT-FFR is the chance for patients to miss the optimal treatment time and delay the revascularization. Therefore, safety concerning patient management with and without CT-FFR was studied. Its impact on patient management and outcome has been studied by Becker et al.^11^ Patients with stenosis of more than 50% in at least 1 coronary artery were assessed. In that study, researchers retrospectively reviewed clinical records. One group of patients underwent CT-FFR analysis, whereas the other group solely underwent CTCA. The clinical decision was made accordingly by cardiologists. Patients had follow up checks 1 year after the initial diagnosis. Figure 2 indicates the basic principle and results of Becker et al,^11^ reaching the following conclusions. First, a greater number of patients received OMT (77.9% in the CT-FFR group; 26.8% in the CTCA group) and refrained from PCI or single-photon emission computed tomography (22.1% in the CT-FFR group; 73.2% in CTCA group) after CT-FFR analysis (*P*<.001). Second, there was no significant difference in major adverse cardiovascular events between the 2 groups of patients in the 1-year follow up. Provided that patients are reevaluated if symptoms persist or worsen within the 1-year timeframe, the CT-FFR analysis would be noninferior to the contemporary patient management strategy. This study’s findings indicate that CT-FFR serves as a reliable and safe method for determining the need for ICA. Although the novel approach maintains patient safety, it could also prevent unnecessary diagnostic examinations. This role of CT-FFR has been supported by many scientific studies worldwide.^12^, ^13^, ^14^ Supplemental Table 3 (available online at https://www.mcpdigitalhealth.org/) summarizes randomized controlled trials that investigated the clinical application of the usual diagnostic pathway and that with CT-FFR.^12^^,^^15^^,^^16^ To ensure the safety and reliability of this novel diagnostic approach, future research could focus on long-term patient survival and complication rate.

Third, CT-FFR could assist cardiologists in revascularization treatment planning in patients with multivessel disease. Depending on the complexity of CAD and clinical background, cardiologists could choose between PCI and coronary artery bypass graft (CABG) as treatment options.^17^ In convention, this decision could be guided by the anatomic SYNTAX score that considers vessel bifurcation, diffuse disease, calcification, thrombus, and total occlusion. Each lesion would be graded and summed up to a total score. A high score indicates a complicated lesion which adds complexity to PCI. It was recommended that a patient with a score greater than 22 should be treated with CABG and vice versa. With CT-FFR, stenosis that is not functionally significant (ie, value of >0.8) would be disregarded in the calculation. The new calculation from anatomic to functional SYNTAX score would reclassify patients’ risk and decide the optimal treatment option.

The modification of the treatment strategy has been proved to be noninferior to the original approach. Guided by FFR, treating patients with PCI or CABG in more complex cases has proved to reduce adverse cardiovascular events, death, and myocardial infarction compared with angiographic guidance.^10^ By recognizing nonhemodynamically substantial stenosis in patients with 3-vessel disease, unnecessary grafts or stent placements could be prevented and surgical time could be shortened. Although the use of FFR in revascularization has reported its benefits in the prevention of adverse cardiovascular events compared with angiography-based alone, performing invasive FFR could be time consuming and tedious. Moreover, CT-FFR could provide a way for cardiologists to plan the treatment. In a multicenter, international, and randomized study investigating treatment decisions on the basis of solely ICA against ICA with the addition of CT-FFR, researchers have documented that CT-FFR modified 18.3% of treatment planning and 6.6% of treatment recommendations.^9^ The use of CT-FFR provides valuable information for treatment planning. This could modify treatment strategy in a significant portion of patients with multiple-vessel disease. Treatment planning using a functional SYNTAX score necessitates FFR, which could be obtained only via ICA traditionally. However, CT-FFR offers a noninvasive alternative, eliminating the need for ICA. Thus, this reduces diagnostic burden by sparing additional examinations and consultations. It could also decrease the fluoroscopy time during ICA, thus reducing the overall radiation dose.

To reiterate, CT-FFR has applications in improving the diagnostic accuracy of CTCA in assessing patients with intermediate stenosis and multiple-vessel disease. It could determine whether the lesion requires revascularization or OMT. In multiple-vessel diseases with complex plaque characteristics, it helps treatment planning and deciding between CABG and PCI. Although all these benefits could also be achieved by ICA and FFR measurements, CT-FFR provides a functional assessment of the ischemia caused by lesions without performing invasive procedures.

## Patient Management with CT-FFR and ITS Impact

**Figure 3:**
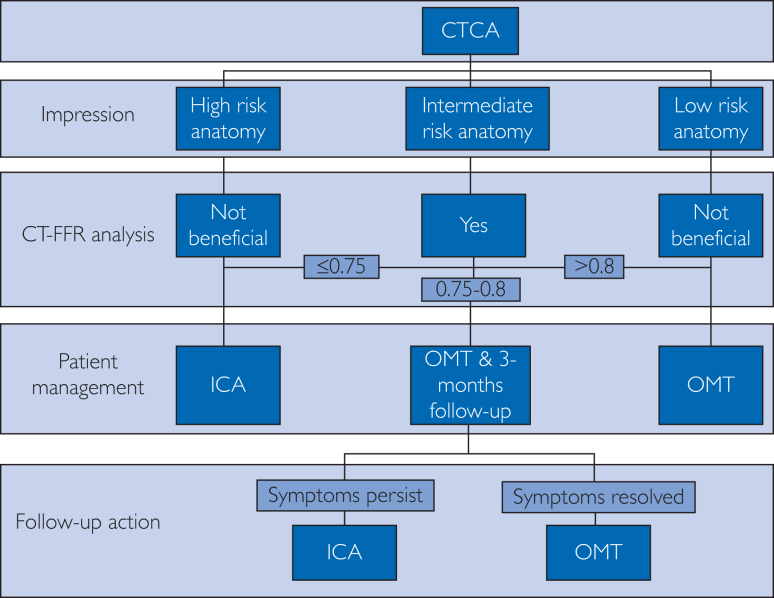
The recommended diagnostic pathway after CTCA.^1^ Flowchart is extracted from Rajiah et al.^1^ CTCA, computer tomography of coronary angiogram; CT-FFR, computer tomography–derived fractional flow reserve; ICA, invasive coronary angiogram; OMT, optimal medical therapy.

Computer tomography–derived fractional flow reserve can be applied on all CTCA cases whenever image quality is optimal for analysis. However, not all patients could benefit from performing CT-FFR. Patients with either low-risk stenosis or high-risk stenosis may not require CT-FFR. Supplemental Table 4 (available online at https://www.mcpdigitalhealth.org/) summarizes the classification of risk. For high-risk anatomy, patients could be referred to ICA and revascularization. In 3-vessel disease, CT-FFR could be useful in treatment planning and may be performed as discussed. For low-risk anatomy, CT-FFR is generally not required. Optimal medical therapy is the recommended patient management.^1^ It was found that among patients with 30%-49% luminal stenosis, 21% exhibited positive CT-FFR results. Conversely, 28% of patients with 71%-90% luminal stenosis found negative CT-FFR results.^18^ Applying CT-FFR in all patients may improve the overall diagnostic accuracy but impose a financial burden on institutions or patients. The recommended diagnostic pathway after CTCA is shown in Figure 3.^1^

In intermediate-risk stenosis, CT-FFR plays a crucial role in differentiating functional stenosis and merely anatomical stenosis. Performing CT-FFR in this group of patients reportedly identified up to 68% more patients who do not experience lesion-specific ischemia in a multicenter study.^1^ The use of CT-FFR in this group of patients could maximize the utility of patients, potentially achieving an optimal balance between financial cost and the effectiveness of the diagnostic pathway. It has been established that refraining from PCI in lesions with negative invasive FFR results was shown to be safe in 15-year follow-ups.^12^ With advancing technology and accuracy, one may anticipate that CT-FFR could play the same role as invasive FFR does.

Computer tomography of coronary angiogram alone would result in additional diagnostic procedures to rule out ischemia, which is an indication of PCI. Conventionally, although this is diagnosed by ICA and FFR, lesion-specific myocardial ischemia could now be identified by CT-FFR with good accuracy. Before planning ICA on patients with suspected CAD, CT-FFR could be performed for screening. PLATFORM study found that screening patients with CT-FFR before referral to ICA led to a significantly lower rate of ICA without obstructive CAD.^12^ Furthermore, CT-FFR provides functional data for decision making on revascularization strategy. These could be beneficial to patients and health care facilities in several ways. First, fewer diagnostic tests would be needed because CT-FFR could be obtained from CTCA data. This could save them from doing extra tests such as single-photon emission computed tomography and ICA, which might cause stress, time expenses, and other medical consultations. Performing less ICA would lead to less radiation and contrast dose to patients. Fewer patients would bear the procedure risk of ICA as well. For health care facilities, fewer diagnostic examinations ease the strain on staff, allowing better allocation of resources and manpower.

One study conducted in the United Kingdom studied the impact of CT-FFR on the financial cost per patient in the National Health Service.^19^ Researchers studied patients who underwent CTCA from 2014 to 2021 and calculated the economic cost until treatment management was reached. The study observed that CT-FFR could shorten the average time of diagnostic investigations (28 days in the CT-FFR group; 44 days in the standard care group without CT-FFR; *P*=.004) at the mean cost of £44.97 (in Great British pounds). In the FORECAST study, cases treated between 2017 and 2019 in the United Kingdom were investigated to find out the medical expenses. It was revealed the total cardiac cost at 9 months between individuals selected for HeartFlow and the standard care group were £1605 and £1491, respectively (*P*=.10).^15^ However, the cost analysis from the FORECAST study may not reflect the current situation. First, there are alternative CT-FFR products available that cost less than HeartFlow. Moreover, different vendors are developing their own CT-FFR products. In the future, the commercial availability of additional products could foster a competitive pricing environment, thereby making it more affordable. This progression is expected to yield benefits for both institutions and patients. Second, the price of HeartFlow for the National Health Service was cut from £700 to £530 after negotiation in 2018.^20^ There would be extra cost savings from implementing CT-FFR nowadays with the FORECAST study as a reference. Third, in the FORECAST study lesions of more than 40% were selected for CT-FFR analysis. Different patient selections for CT-FFR would affect the overall cost of this diagnostic pathway. Graby et al^19^ reported that stenosis of more than 50% was the most cost effective and safe patient selection criteria for CT-FFR analysis.^19^ This paves the way for a potential research topic investigating the optimal patient selection criteria in Hong Kong. Moreover, CT-FFR could save patients time as additional examinations are usually not required. Implementing CT-FFR in the diagnostic pathway might alleviate the pressure in the overloaded public health care system in Hong Kong.

## Limitation of the Technology

There are situations in which CT-FFR may not provide an accurate functional assessment of the stenosis. One of them is the calcium burden of coronary arteries. Coronary artery calcium could be quantified by the Agatston score, which reflects the volume of calcification. Although CT-FFR has been shown to perform better than CTCA alone regardless of the Agatston score, a decrease in AUC was observed in the high Agatston score range (>400).^21^^,^^22^ It is believed that extensive calcification imposes a negative impact on identifying the vessel lumen and its course. In scenario when the calcium blooming is significant, the CT-FFR analysis may be rejected. Certain algorithms that could maintain the diagnostic performance in cases with high Agatston scores were reported.^7^^,^^10^ However, these algorithms are still under research and development. To obtain functional information in the presence of extensive calcification, CT-FFR may not be the best option and alternative imaging tests should be considered.^20^ Stress echocardiography, for example, could also provide functional information for treatment decisions. Therefore, the use of CT-FFR in patients with heavy coronary calcification should be carefully considered before this area is well investigated.

The use of CT-FFR may be limited to coronary arteries with the metallic stent. The major challenge in this scenario is the blooming caused by the metallic stent. This could cause an artifactual narrowing of the lumen.^22^ This affects the accuracy of CT-FFR analysis because it depends on luminal diameter. For this reason, the assessment of the stent and its remaining course may be limited. One study investigating its use on in-stent restenosis (ISR) mentioned the challenge in post-PCI cases.^23^ In-stent restenosis is the major complication of PCI. Post-PCI CTCA may be done to rule out ISR if clinically indicated. Assessing stents of small caliber could be more challenging because a higher proportion of stent lumen is within the blooming artifact. It was shown that CT-FFR reported an accuracy of 85.7% in diagnosing ISR, given manual editing of the luminal border and stenosis boundary.^24^ The reported average time for manual editing was 33.5 minutes. On the contrary, evidence reported that ISR could be diagnosed using CTCA only with an accuracy of 94.1%, particularly with a more advanced CT scanner. A higher accuracy could be achieved by using an advanced iterative reconstruction algorithm, stent kernel, and integrated circuit detector, which improves spatial resolution.^25^ Although both methods demonstrate sufficiently good diagnostic performance, the extra time and financial cost from CT-FFR and manual editing might limit the practical application. Nevertheless, this technique could be further researched as a solution to the artifactual narrowing problem. Note that CT-FFR could still be applied on coronary vessels without a metallic stent in its whole course.

There are several patient-related scenarios where the use of CT-FFR is limited. One study indicated that recent ST-elevation myocardial infarction (STEMI) within the past 1 month is negatively associated with CT-FFR accuracy.^26^ This phenomenon could be attributed to several factors. First, patients who have experienced a myocardial infarction may exhibit a diminished response to vasodilators for up to 6 months after infarction. Second, during the recovery period after STEMI, patients often engage in less physical activity. This reduction in activity can lead to decreased coronary blood flow and oxygen demand, potentially triggering epicardial vasoconstriction.^27^ The combination of these factors may result in a reduced coronary arterial volume-to-myocardial mass ratio during the post-STEMI recovery phase. Altering the FFR measurement, these considerations highlight the limitation CT-FFR in specific patient populations, particularly those recovering from recent myocardial infarction.

**Figure 4:**
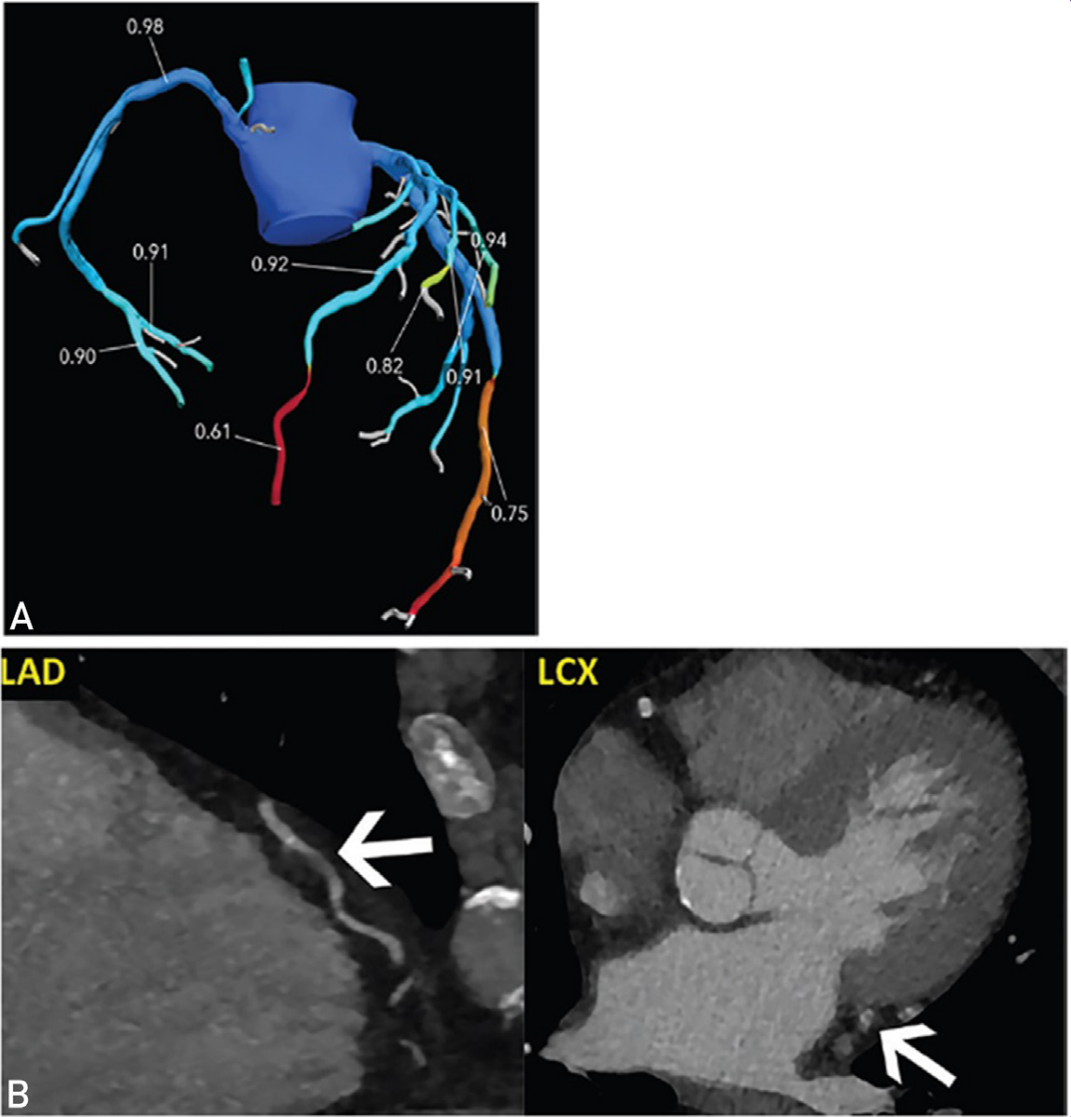
Suboptimal image quality causing artifactual CT-FFR results.^1^ A, CT-FFR image of a 73-year-old man showing positive results in LAD and LCx. B, Motion artifacts are found in LAD and LCx. These account for abnormal CT-FFR values. Pictures are adapted from Rajiah et al.^1^ CT-FFR, computer tomography–derived fractional flow reserve; LAD, left anterior descending artery; LCx, left circumflex artery.

Another limitation of applying CT-FFR is that optimal image quality is required. Suboptimal image quality could be attributed to breathing artifacts, cardiac motion, prominent image noise, and metallic artifacts. Motional artifacts caused by cardiac or respiratory motion could result in abnormal CT-FFR values. In Figure 4, the motion artifact could be seen at left circumflex artery, which mimicked a focal stenosis. Analyzed by HeartFlow, the software returned a value of 0.61 in the distal segment. Whether this is a genuine lesion is doubtful without another set of clearer CTCA images. It was reported that the motion artifact was the major contribution to the rejection of CT-FFR analysis.^28^ Performing analysis using HeartFlow could be challenging in scenarios when the patient could be uncooperative. This could lead to poor image quality and inaccurate CT-FFR results due to image artifacts.

**Figure 5:**
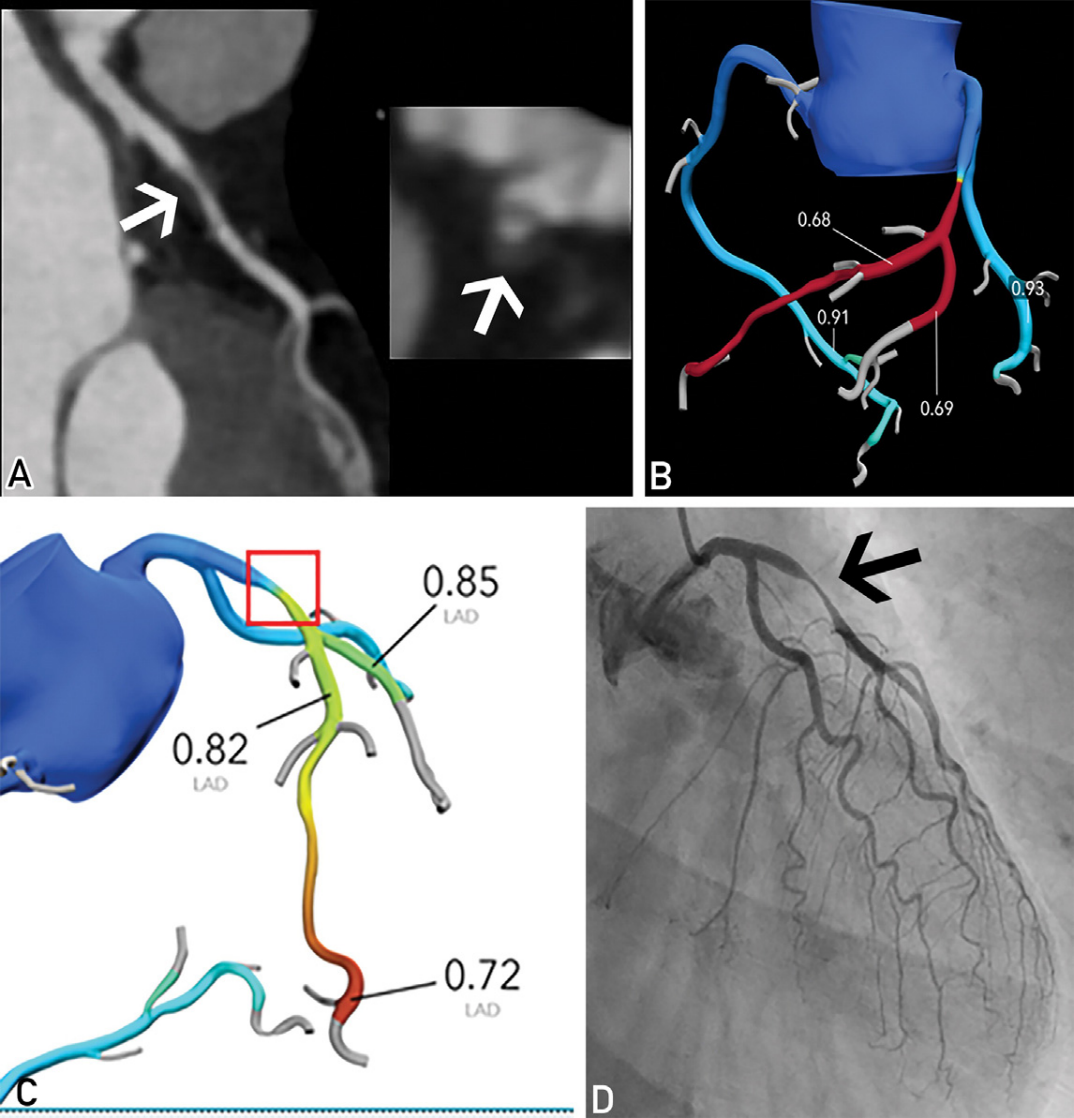
Segmentation error causing abnormal CT-FFR results.^1^ A, Curved multiplanar reconstruction and axial image show severe stenosis of mid-LAD. B, Abnormal CT-FFR value is noted in LAD distal to the lesion. The modeling of the narrowed LAD segment is tighter than what is observed in the CTCA image. C, CT-FFR image after correction in modeling the LAD segment. The CT-FFR value distal to the lesion is 0.82. D, ICA image shows a severe stenosis in mid-LAD. The invasive FFR value was 0.8, complying with the CT-FFR results. Pictures are adapted from Rajiah et al.^1^ CT-FFR, computer tomography–derived fractional flow reserve; ICA, invasive coronary angiogram; LAD, left anterior descending artery.

Segmentation errors could also result in misleading CT-FFR values. When the modeling of the coronary artery is tighter than it should be, the extent of stenosis could be exaggerated. This might cause an artifactual decrease in CT-FFR value. In Figure 5, the left anterior descending artery segmented by the software appeared narrower than that in the CTCA image. The erroneous exclusion of the area of interest was corrected and processed by HeartFlow. The CT-FFR value of the poststenotic segment changed from 0.68 to 0.82, which indicated a different diagnostic conclusion. Invasive FFR was found to be 0.8, which is consistent with the CT-FFR result. Inaccurate recognition of coronary arteries could also result in missing functional stenosis in small branches. In these scenarios, it is recommended to correlate the original CTCA images and rule out the possibility of artifacts.^1^ Possible remedies include image reconstruction and remodeling of the affected segment.

The reconstruction algorithm and image noise level might affect the performance of CT-FFR analysis. Using iterative reconstruction instead of filter back projection, the average time to process by the ML-based model could be shortened by 20%.^29^ The major effect of iterative reconstruction is to suppress image noise. Faster processing of CT-FFR could contribute to improved segmentation capability and less manual correction during modeling. The absence of a novel iterative reconstruction algorithm in old systems may necessitate increased time and manpower to conduct CT-FFR analysis effectively. Another parameter affecting the performance of CT-FFR analysis is the image kernel. A sharper reconstruction filter was reported to yield a better accuracy of 91% compared with 82% in a medium-smooth kernel using Siemens Somatom Force (*P*=.02).^30^ Given the variability in image texture resulting from reconstruction by disparate imaging systems and parameters, there may be consequential differences in diagnostic accuracy.

Another limitation of the technology is the long processing time of CT-FFR. HeartFlow is the only CFD model that obtained FDA clearance in 2024. The image processing time extends to several hours, and the service efficiency during nonoffice hours is subject to variability. In the future, alternative solutions using ML could solve the issue because it could be run onsite by the technologist.

## The Future Development of CT-FFR

Upcoming advancements are anticipated to leverage big data and advanced deep learning techniques to enhance the synthesis of a thorough functional and anatomical evaluation derived from CTCA, enabling a parametric determination of CAD severity. Because the turnaround time could be long using model-based HeartFlow, the faster ML-based software could overcome this issue. DEEPVESSEL FFR, invented by Keya Medical Technology, claimed that the average computational time of each patient was 2 minutes.^9^ This deep learning approach, like HeartFlow, could also save time for manual segmentation and regions of interest extraction. Images obtained from the CT scanner could be directly uploaded to the online platform, saving additional costs for local processing devices.

Besides the deep learning approach to shorten the computation time, an attempt to simplify CFD computation has been reported; uCT-FFR is one of the algorithms using the computation of CFD like HeartFlow does. It simplifies the process in 3 ways. First, anatomy segmentation is done by a convolutional neural network that is claimed to be more accurate and require less manual editing. Second, a larger mesh element is assigned to the aorta to reduce computational load. Third, the concept of transluminal attenuation coefficient is introduced into the derivation of the boundary condition in solving the CFD equation. It is a linear regression coefficient relating to the intraluminal Hounsfield unit and distance from the coronary ostium. It was reported that the whole computation process could be completed in minutes.^10^ Moreover, the computation could be completed on a standard desktop instead of a supercomputer as HeartFlow does. Another approach to simplify the computation is to use a reduced-order model. Instead of solving 3D CFD equations, a modified 1D computation is used. This was found to be more efficient with similar results.^31^ Strategies to accelerate the computation have been proposed and researched. In the future, more commercial products will be released. This could provide the patients and institutions with a faster, more accurate, and affordable solution.

## Potential Competing Interests

The authors report no competing interests.
