# Supplementary material for: Clinical Applications of Fractional Flow Reserve Derived from Computed Tomography in Coronary Artery Disease
Source: Mayo Clin Proc Digit Health. 2024 Dec 14;3(1):100187. doi: 10.1016/j.mcpdig.2024.100187 (PMC11975968; doi:10.1016/j.mcpdig.2024.100187)
Supplement: Supplemental Figure and Tables [file mmc1.docx]

Supplementary Figure


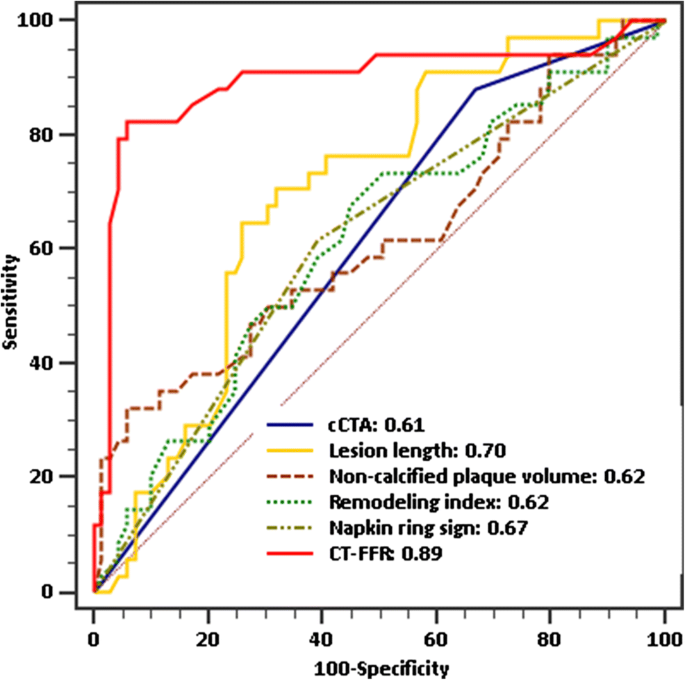


**Supplementary Figure 1**. ROC Curve of Diagnostic Performance of Plaque Characteristics and CT-FFR^5^. cCTA: Coronary Computed Tomographic Angiography. The ROC plot is adapted from von Knebel Doeberitz et al. (2019).

Supplementary Tables

| Parameter | Sensitivity | Specificity | PPV | NPV |
| --- | --- | --- | --- | --- |
| Lesion Length > 21mm | 71% (53-85%) | 68% (56-79%) | 52% (42-62%) | 83% (73-89%) |
| Non-calcified plaque volume > 132mm^3^ | 62% (44-78%) | 65% (53-76%) | 47% (37-57%) | 78% (69-85%) |
| Remodeling index > 1.03 | 74% (56-87%) | 49% (37-62%) | 42% (34-49%) | 79% (67-87%) |
| Napkin-ring sign | 74% (56-87%) | 59% (47-71%) | 47% (39-56%) | 82% (72-89%) |
| Stenosis $\boldsymbol{\geq}$ 50% | 88% (73-97%) | 33% (22-46%) | 40% (35-45%) | 85% (68-94%) |
| *CT-FFR $\boldsymbol{\leq}$ 0.8 | 82% (66-93%) | 94% (86-98%) | 88% (73-95%) | 92% (84-96%) |

**Supplementary Table 1.** Diagnostic performance of plaque characteristics and CT-FFR in lesion-specific ischemia^5^. *Note that CT-FFR demonstrates better specificity and PPV among all features. PPV: positive predictive value, NPV: negative predictive value. 95% confidence intervals are shown in parentheses.

| Algorithm | Year of the Publishment | Sensitivity (%) | Specificity (%) | PPV (%) | NPV (%) | AUC | Note |
| --- | --- | --- | --- | --- | --- | --- | --- |
| HeartFlow^8^ | 2021 | 87 (82-92) | 73 (67-79) | 75 (69-81) | 86 (80-91) | 0.85 | This software obtained FDA approval in 2022. Vessels having stenosis within the range of 50%-90% were included in the analysis. The diagnostic performance shown here is on a per-vessel basis. |
| DEEPVESSEL FFR^9^ | 2019 | 98 (87-100) | 77 (58-90) | 85 (72-94) | 96 (79-100) | 0.93 | This software obtained FDA clearance in 2022. Vessels having stenosis within the range of 30%-90% were included in the analysis. The diagnostic performance shown here is on a per-vessel basis. |
| Siemens cFFR (Machine Learning)^4^ | 2018 | 81% (75-86) | 76% (71-81) | 70% (64-76) | 85 (81-90) | 0.84 | This software is not commercially available. Only lesions >50% stenosis were analyzed. The diagnostic performance shown here is on a per-vessel basis. |
| Siemens cFFR (CFD based)^4^ | 2018 | 82 (77-87) | 76 (71-82) | 70 (64-76) | 86 (82-90) | 0.84 | This software is not commercially available. Only stenosis >50% were analyzed. The diagnostic performance shown here is on a per-vessel basis. |
| General Electric xFFR version 1.4^7^ | 2023 | 93 (77-99) | 100 (90-100) | 100 (100-100) | 95 (82-99) | 0.98 | It is a prototype software currently under development. Stenosis <50% was also included in the data analysis. |
| uCT FFR^10^ | 2020 | 89 (84-94) | 91 (88-95) | 86 (80-91) | 94 (90-97) | 0.92 | It is a prototype software currently under development. Stenosis within the range of 20%-90% was included in the analysis. The diagnostic performance shown here is on a per-vessel basis. |

**Supplementary Table 2.** The diagnostic performance of common CT-FFR algorithms. The data is expressed in percentages. 95% confidence intervals are shown in parentheses.

| Clinical Trial | Year of Publishment | Recruiting Sites | Randomisation Group for Subjects | Highlights |
| --- | --- | --- | --- | --- |
| PLATFORM^12^ | 2015 | Europe | 1. CTCA±CT-FFR vs *Non-invasive test 2. CTCA±CT-FFR vs Initial ICA | 1. No significant difference in the rate of ICA was observed between CTCA±CT-FFR and the non-invasive test group  2. No significant difference in the rate of false positives (obstructive CAD diagnosed in initial testing) was observed between CTCA±CT-FFR and the non-invasive test group 3. CT-FFR led to the cancellation of ICA in 61% of the referral. |
| FORECAST^15^ | 2021 | United Kingdom | CTCA±CT-FFR vs *Non-invasive test/Initial ICA | 1. No significant difference in mean total cardiac cost over 9 months between 2 groups 2. No significant difference in symptoms and MACE between 2 groups 3. CTCA+-CT-FFR reduced the referral to ICA  4. Less non-obstructive CAD is observed in ICA among subjects in the CTCA+-CT-FFR group |
| TARGET^16^ | 2023 | China | CTCA±CT-FFR vs Stress test/ Exercise ECG/Perfusion scan | 1. Results are generally consistent with the FORECAST study 2. For patients referred to ICA after initial testing, less proportion of patients who showed non-obstructive CAD was observed in the CT-FFR group 3. The revascularisation rate in the CT-FFR group was higher within 90 days but similar within 1 year after the first ICA |

**Supplementary Table 3.** Randomized Clinical Trials comparing diagnostic pathways using CT-FFR versus that of the usual care strategy. *Note that non-invasive test includes stress echo, perfusion scan, stress MRI, exercise ECG, and CTCA

| Affected Segment | High Risk | Intermediate Risk | Low Risk |
| --- | --- | --- | --- |
| Left Main Stem | ≥50% | 30%-49% | ≤30% |
| LAD | ≥70% | 30-69% | ≤30% |
| Other coronary vessels | Three-vessel stenosis; Complete occlusion | ≥70% | <70% |

**Supplementary Table 4.** The definition of high-risk, intermediate-risk and low-risk anatomy in relation to affected coronary artery and degree of stenosis. LAD: Left anterior descending artery. Other coronary vessels include the right coronary artery (RCA), left circumflex artery (LCx) and their branches.
